# Proteome analysis of the hyaluronic acid-producing bacterium, *Streptococcus zooepidemicus*

**DOI:** 10.1186/1477-5956-7-13

**Published:** 2009-03-28

**Authors:** Esteban Marcellin, Christian W Gruber, Colin Archer, David J Craik, Lars K Nielsen

**Affiliations:** 1 Australian Institute for Bioengineering and Nanotechnology (AIBN), University of Queensland, QLD 4072, Australia; 2 Institute for Molecular Bioscience (IMB), University of Queensland, QLD 4072, Australia; 3 Center for Biomolecular Medicine and Pharmacology, Medical University of Vienna, Waehringer Str. 13a, A-1090 Vienna, Austria

## Abstract

**Background:**

*Streptococcus equi *subsp. *zooepidemicus *(*S. zooepidemicus*) is a commensal of horses and an opportunistic pathogen in many animals and humans. Some strains produce copious amounts of hyaluronic acid, making *S. zooepidemicus *an important industrial microorganism for the production of this valuable biopolymer used in the pharmaceutical and cosmetic industry. Encapsulation by hyaluronic acid is considered an important virulence factor in other streptococci, though the importance in *S. zooepidemicus *remains poorly understood. Proteomics may provide a better understanding of virulence factors in *S. zooepidemicus*, facilitate the design of better diagnostics and treatments, and guide engineering of superior production strains.

**Results:**

Using hyaluronidase to remove the capsule and by optimising cellular lysis, a reference map for *S. zooepidemicus *was completed. This protocol significantly increased protein recovery, allowing for visualisation of 682 spots and the identification of 86 proteins using mass spectrometry (LC-ESI-MS/MS and MALDI-TOF/TOF); of which 16 were membrane proteins.

**Conclusion:**

The data presented constitute the first reference map for *S. zooepidemicus *and provide new information on the identity and characteristics of the more abundantly expressed proteins.

## Background

*Streptococcus equi *subsp. *zooepidemicus *(*S. zooepidemicus*) is a commensal of the upper respiratory tract, skin and urogenital tract of horses, some other animals and humans. It is a primarily opportunistic pathogen in many animal species, including domesticated animals such as horses, cows, pigs, sheep, and dogs. It is the primary cause of equine respiratory tract infections in foals and the leading cause of infertility in mares [1,2]. Sequencing of strain H70 by the Sanger Institute was sponsored by The Horserace Betting Levy Board in UK.

*S. zooepidemicus *also infects humans, typically through zoonotic transmission from domesticated animals or by ingestion of improperly pasteurised dairy products [3]. Clinical manifestations in humans include mild respiratory disease, pneumonia, endocarditis, endophthalmitis, septic arthritis, meningitis, septicemia, streptococcal toxic shock syndrome and post-streptococcal glomerulonephritis. A large outbreak with 253 cases of acute nephritis in Brazil was linked to the consumption of unpasteurised cheese and a strain isolate, MGCS10565, was recently sequenced [4].

Lancefield Group A and C streptococci produce the exopolysaccharide hyaluronic acid (HA). The HA capsule protects streptococci from phagocytosis [5-8] and may facilitate tissue adherence and invasion [5,9]. The HA capsule is an important virulence factor for *S. pyogenes *and disruption of the *has *operon results in a significant reduction in pathogenicity [10,11]. Similarly, the HA capsule significantly enhances virulence of *S. equi *subsp. *equi *(*S. equi*), a pathogenic biovar of *S. zooepidemicus *that causes strangles in horses [8,12]. Whereas virulent isolates of *S. equi *are generally highly encapsulated, capsule synthesis is highly variable in *S. zooepidemicus *and usually lost following primary culture, suggesting that encapsulation is under regulation by host factors [13]. In *S. pyogenes*, HA production as well as other virulence factors, are regulated by the CsrRS protein (also known as CovRS). CsrRS is a two-component regulator of extracellular virulence factors in Group A streptococcus [14,15] and can be induced by a human antimicrobial peptide [16].

The strain used in this study, ATCC 35246, was isolated during 1975 Sichuan province swine streptococcosis outbreak, in which over 300,000 pigs died [17,18]. ATCC 35246 is a highly encapsulated *S. zooepidemicus *strain with a stable mucoid phenotype even in the absence of selection [19]. Non-mucoid variants generated in continuous culture on complex medium are similarly stable in serial passage without selection, but revert to a mucoid phenotype when exposed to horse serum [19].

The objective of this study was to develop a proteomics protocol and a reference map. The results can be used for comparative analysis of mucoid and non-mucoid strains, in order to better understand the role of HA regulation in *S. zooepidemicus *virulence, facilitate the design of better diagnostics and treatments [20], and guide engineering of superior HA production strains [21].

Despite advances in mass spectrometry-based techniques for proteomics analysis, two dimension gel electrophoresis (2-DE) is still the most commonly used protein separation technique, especially with the use of new fluorescent dye systems which enable differential gel electrophoresis (2-D DIGE) [22]. 2-DE-based proteome analyses have been performed for many microorganisms including the closely related *S. mutans *[23], *S. pneumoniae *[24], *S. thermophilus *[25,26], *S. pyogenes *[27-31] and more recently *S. suis *[32]. There is currently no reference map for *S. zooepidemicus*, though an extracellular immunoproteome map was recently reported for ATCC 35246 [18].

Obtaining high quality 2-DE gels and harvesting protein extracts from encapsulated bacteria has proven to be technically challenging [24]. *S. zooepidemicus *ATCC 35246 produces very large amounts of high molecular weight HA, which presents several technical challenges not found in other streptococci: first, the buoyancy of HA limits the formation of a cellular pellet by centrifugation; second, the complex structure of the cell envelope enables the cell to resist conventional lysis methods [24]; and thirdly, HA interferes with standard protein extraction techniques.

In this paper we present a novel method that combines capsular removal using enzymatic digestion (hyaluronidase) with mechanical cell lysis, followed by protein extraction and a precipitation technique that minimizes protein loss. Proteins resolved from *S. zooepidemicus *strain ATCC 35246 were further identified using LC-MS/MS and MALDI-TOF/TOF to create the first reference map compatible with DIGE of any streptococcal species.

## Results and discussion

### Hyaluronidase treatment combined with optimised extraction and precipitation techniques yields high-quality proteomic gels

**Figure 1:**
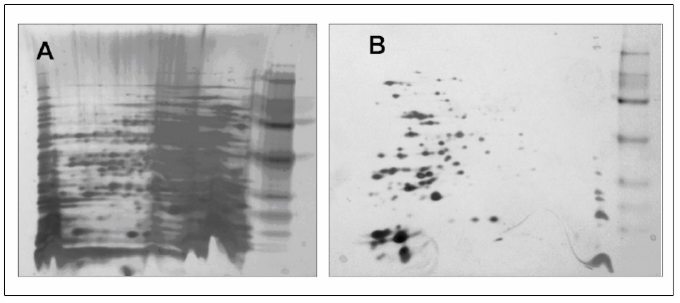
**2-DE gels of *S. zooepidemicus*. **Proteins were separated using pH gradient 4–7 on 7 cm 12% polyacrylamide gels. Following separation, proteins were stained with silver nitrate. When HA was not removed, poor quality, streaky gels were obtained (A); digestion of HA prior to protein extraction resulted in high quality, streak-free gels (B).

Proteomic investigation of HA-encapsulated bacteria is problematic because HA buoyancy limits the formation of a cellular pellet by centrifugation and residual HA in protein extractions can interfere with subsequent analyses. In HA-contaminated samples, protein quantification by Bradford assay and 2D-Quant (GE Healthcare) were inaccurate, resulting in overloaded gels. HA also interfered with iso-electric focusing and poor quality 2-D gels were observed even after protein precipitation (Figure 1A). In solution, HA folds into a helical structure formed by hydrogen bonds between carboxyl groups and N-acetyl groups [33]. These hydrogen bonds form protein aggregates and secondary structures that interfere with the mobility in the gel; therefore, in our samples it was necessary to remove HA prior to protein extraction. The use of hyaluronidase to remove the HA capsule prior to protein extraction allowed accurate protein quantification using 2D-Quant and improved iso-electric focussing.

Surface proteins are often immunogenic [30,31,34], and are important in a proteome study. However, a lysis buffer designed to isolate streptococcal membrane proteins [24,35] (lysis buffer B) did not effectively denature proteins and gave poor DIGE results (data not shown). Therefore, a conventional lysis buffer (buffer A), which is compatible with DIGE, was used. In combination with hyaluronidase treatment, the conventional lysis buffer A allowed us to obtain high quality gels suitable for proteomic analysis (Figure 1B).

**Figure 2:**
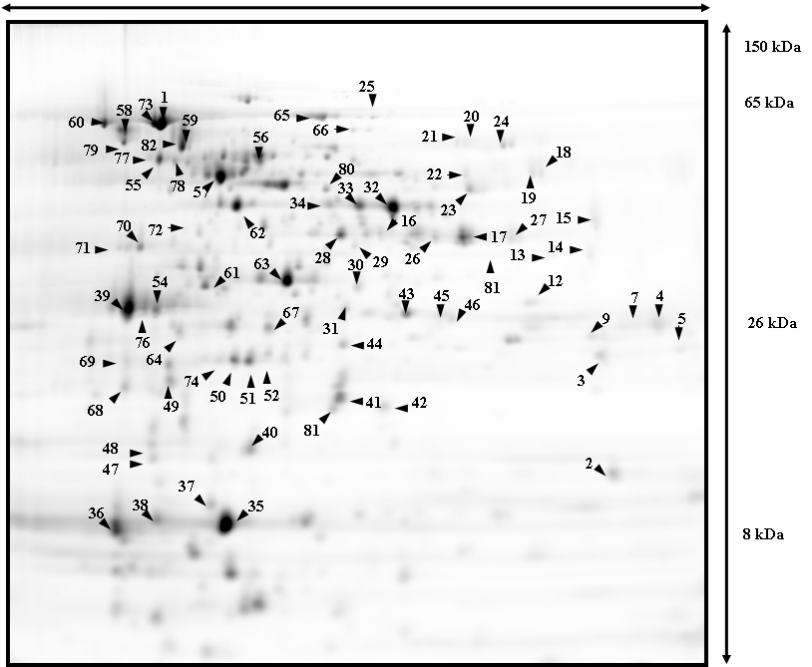
**Reference map of *S. zooepidemicus *(ATCC 35246)**. Proteins were harvested using hyaluronidase to remove the HA capsule. Proteins were separated using pH gradient 4–7 and 24 cm 12% polyacrylamide gels. Proteins were labelled with cy3 and visualised using a typhoon scanner. Protein spots identified using LC-ESI-MS/MS and MALDI-TOF/TOF numbered and listed in Additional file 1. Numbers not listed in the Additional file did not yield an MS-identified protein hit.

Samples for 2-DE should also be free of contaminants (salts, ionic detergents, lipids, polysaccharides, DNA and RNA). Furthermore, for 2-D DIGE, high protein concentrations are required. Protein precipitation has proven to be a valuable tool to concentrate samples and remove impurities; however, it is necessary that the precipitation technique does not cause artifacts such as variable protein losses resulting in a change in relative abundance of proteins. We compared precipitation protocols based on acetone or TCA as well as a commercial precipitation kit (2-D Clean-Up; GE-Healthcare). The best results were obtained with the 2-D Clean-Up kit; we observed a two-fold increase in protein recovery, clearer gels and better-resolved spots compared to the other techniques (data not shown). The combination of hyaluronidase treatment, conventional lysis (using buffer A) and 2-D Clean-Up allowed for separation of 682 proteins of *S. zooepidemicus *(Figure 2). The procedure was also reproducible between replicate extractions, showing only slight variation in spot numbers detected on each gel (625,622 and 619). Resulting protein spots were also compatible with DIGE and MS identification techniques (see below). This protocol can be used for comparative analysis of mucoid and non-mucoid strains, in order to elucidate the role of HA in *S. zooepidemicus *virulence.

### Reference Map of the *S. zooepidemicus *proteome

**Figure 3:**
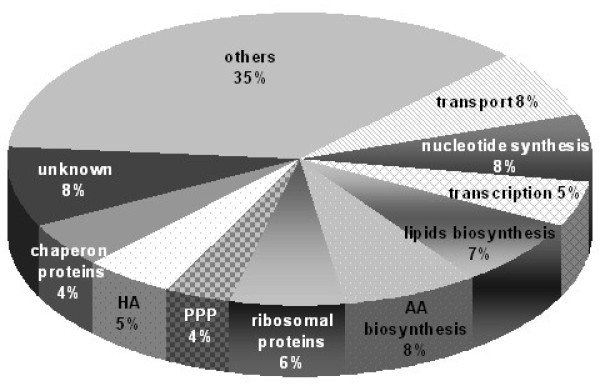
**Functional grouping of proteins identified from *S. zooepidemicus *proteome**. Proteins from the glycolysis, pentose phosphate, and HA pathways were identified in the reference map. Biomass constituent enzymes such as amino acid, lipids and peptidoglycan synthesis were also identified. HA: hyaluronic acid, PPP: phosphate pentose pathway; AA: amino acid.

Protein reference maps have proven to be valuable tools to investigate protein expression in cells, tissues and whole microorganisms. After obtaining good quality 2-DE gels, proteins need to be identified and MS remains the best technique to achieve this. The most abundant spots in the gel were digested with trypsin and analysed with LC-ESI-MS/MS. Where LC-ESI-MS/MS identified proteins with less than our threshold of 99% confidence, results were confirmed by MALDI-MS-TOF/TOF. From the 682 visualised proteins, a total of 86 proteins were identified from the reference map in 73 spots; 18 spots contained more than one protein and eight proteins were identified in multiple spots (Figure 2). The number of identified proteins is similar to the 77 proteins recently identified in the reference map for *S. suis *[32]. Enzymes were grouped according to source pathway (glycolysis, pentose phosphate, and HA pathways) or biomass product type (amino acids, lipids and peptidoglycan synthesis) (Figure 3). Identified proteins included four enzymes of the HA pathway: UDP-N-acetyl-glucosamine pyrophosphorylase (EC 2.7.7.23; Spot 20), UDP-glucose pyrophosphorylase (EC 2.7.7.9; Spot 31), phosphoglucosamine mutase (EC 5.4.2.10; Spot 55) and UDP-glucose dehydrogenase (EC 1.1.1.22; Spot 80 and 34).

Good proteome reference maps are needed because *in silico *predictions fail to predict post-translational modifications, proteolyic cleavage and non-ideal electrophoretic separation. Eight proteins were identified in multiple spots (195978702, 195977350, 195978812, 19597756, 195977347, 195978812, 195977545, 195977347). Such shifts can be caused by modifications such as phosphorylation, glycosylation or acetylation, which can alter molecular weight and/or p*I*. For all identified proteins, theoretical p*I *and molecular masses were calculated using an ExPASy tool and compared with experimental data. The variation between predicted and measured p*I *was less than 0.5 for most proteins with greater p*I *variation seen towards the most basic region of the gel. The variation between calculated molecular mass and experimental data was low (within 10%) with the greatest variation observed for masses greater than 55 kDa. It is possible that some of the observed differences are due to genomic variations between MGCS10565 and ATCC 35246.

Seven of the proteins isolated were identified as hypothetical proteins. Using InterProScan Sequence Search (EMBL-EBI; ) to identify protein properties (families, domains, repeats and sites), one protein was identified as belonging to a phosphotransferase system (195977564) and another containing the catalytic region of a diacylglycerol kinase (195978835). The function of the other proteins remains unknown; localising these proteins on our reference map may contribute to future understanding of their function.

Curiously, one of the proteins isolated (spot 2; Additional file 1) could not be identified in the *S. zooepidemicus *genome. When BLASTed against the Swiss Prot database, the protein was most closely related to a Cu-Zn superoxide dismutase (SOD) (EC 1.15.1.1) from *Bos mutus grunniens*. Identification was confirmed by MALDI-MS-TOF/TOF. We suspected this to be a contamination from the hyaluronidase that was used to digest HA, which was derived from a bovine source. Upon further review, two bands were observed in SDS-PAGE of the hyaluronidase and MS identification of the second band confirmed that the SOD was a contaminant from the hyaluronidase preparation (data not shown).

### Surface proteome

The surface proteome is important for the identification of vaccine candidates and virulence factors involved in invasive infection [20,36]. In the complete *S. zooepidemicus *genome MGCS10565, 45 (2%) cell wall proteins, 1002 (44%) cytoplasmic proteins, 379 (16%) cytoplasmic membrane proteins, 185 (7%) extracellular proteins and 703 (31%) undetermined localization proteins were identified using the PSORTb 2.0 algorithm [37]. Of the 86 proteins identified in this study, 16 were predicted to be membrane proteins and two to be extracellular proteins (Additional file 1).

The surface proteome of *S. zooepidemicus *ATCC 35246 has recently been assessed for immunogenicity [18]. 2-DE gels of extracted extracellular proteins were blotted with convalescent sera raised in *S. zooepidemicus*-immunized SFP minipigs and eight immunoreactive proteins identified. Of these eight proteins, four were also identified in our analysis: phosphoglycerate kinase (EC 2.7.2.3; PGK; Spot 34), UDP-N-acetyl-glucosamine pyrophosphorylase (E2.7.7.23; Spot 20), UDP-glucose pyrophosphorylase (EC 2.7.7.9; Spot 31) and phosphofructokinase (EC 2.7.1.11; Spot 28). Moreover, the strain has also recently been used to develop a vaccine that protected mice [38] and the M-protein of *S. zooepidemicus *has been found to be distinct from other subspecies [17].

Seventeen of the proteins identified in our study were also found in the extracted surface proteome of *S. pyogenes *[29]. This represent a quarter of the 66 identified surface proteins with known function and includes 10 out of the 33 immunoreactive proteins. Among these, the two heat shock proteins – DnaK (Spot 1 and 60) and GroEL (Spot 59 and 82) – have previously been shown to be immunogenic for *S. pyogenes *[39,40] and were also identified in the extracted surface proteome of *S. agalactiae *[41]. Phosphoglycerate kinase (EC 2.7.2.3; Spot 34) was also identified in the surface proteome of *S. agalactiae *and anti-sera against phosphoglycerate kinase offers a degree of protection in a neonatal mice model [41].

2-DE based proteomics – whether based on whole-cell or surface extraction – is not ideal for the identification of surface proteins, due either to hydrophobicity and aggregation during IEF, or their low abundance [20]. Some of the surface exposed proteins of *S. zooepidemicus *have proline-rich repeats that hinder migration in gels. Moreover, extracted surface proteomes suffer from contamination with abundant cytosolic proteins. Indeed, only 7 of the 66 identified surface proteins in the extracted surface proteome of *S. pyogenes *had identifiable signal peptides [29]. In contrast, when the *S. pyogenes *surface proteome was explored by protease treatment of whole-cells and identification of resulting peptides, only 4 of 72 proteins were identified as cytoplasmic [30].

None of the reported *S. zooepidemicus *surface proteins ZAG, FNZ and SzP previously considered as vaccine components [17,42,43] were identified in the extracellular immunoproteome of *S. zooepidemicus *ATCC 35246 [18] or in our study. The 2-DE protocol developed here for whole-cell extracts should be complemented with a more targeted surface peptide MS method for a complete study of virulence factors and identification of immunogenic proteins capable of contributing to acquired protective immunity.

## Conclusion

Presented here is the first proteomic reference map of an important pathogenic microorganism, *S. zooepidemicus*. We have developed an optimised protocol for HA removal, cell lysis, protein precipitation and solubilisation; the protocol is compatible with DIGE and MS. A comparison with the extracellular immunoproteome of *S. zooepidemicus *and *S. pyogenes *suggests that the protocol can be used to identify immunogenic membrane proteins of potential use in vaccines. Both 2-DE approaches, however, suffer from a bias towards abundant membrane proteins and possible contaminants and fail to identify low-abundant, strongly immunogenic surface proteins such as SzP.

The protocol will find immediate use in the comparison of mucoid and non-mucoid strain variants of *S. zooepidemicus*, which will help to better understand the role of HA regulation in *S. zooepidemicus *virulence as well as for the design of superior production strains for HA production. Four enzymes from the HA pathway were mapped in this study: UDP-N-acetyl-glucosamine pyrophosphorylase (two copies), phosphoglucosamine mutase, glucose-6-phosphate isomerase and UDP-glucose dehydrogenase. The use of this reference map for metabolic engineering of the strain is currently ongoing in our laboratory, and has resulted in a provisional patent. Results of further studies will be reported in the near future.

## Methods

### Strain culture

The mucoid Group C, *S*. *zooepidemicus *strain ATCC 35246 was obtained from the American Type Culture Collection (Rockville, MD, USA). To ensure the mucoid phenotype, cultures were grown in sheep blood agar and transferred into a chemically defined medium modified from [44] by adding 20 g L^-1 ^of glucose, 4.5 g L^-1 ^of acetate and 50 mg L^-1 ^of uridine. Growth experiments were conducted in a 2 L bioreactor (Applikon) at a working volume of 1.4 L and 37°C. The reactor was agitated at 300 rpm and anaerobic conditions were maintained by top sparging with nitrogen during fermentation. The pH was controlled at 6.7 by addition of 5 M NaOH.

### Lysis buffer

Cells were grown to exponential phase (OD_530 _= 2–4). In some cases, hyaluronidase (10 mg per 100 mL) was added and the cells incubated at 37°C for 10 min. Cells with hyaluronidase treatment were centrifuged at 20,000 × *g *for 20 min at 4°C and the pellet resuspended in 30 mL lysis buffer A or B. Cells without hyaluronidase treatment were centrifuged at 50,000 × *g *for 20 min at 4°C. Because of its rheological properties and buoyancy, cells surrounded by HA need a higher centrifugal force than cells treated with hyaluronidase. Subsequently, the pellet was resuspended in 30 mL wash buffer (50 mM NaH_2_PO_4_, 5 mM EDTA, 10% (v/v) glycerol, pH 7) to remove any residual medium. Cells were then pelleted again and resuspended in 5 mL lysis buffer A or B. Lysis buffer A is a standard lysis buffer consisting of 30 mM Tris, 7 M urea, 2 M thiourea, 4% CHAPS and protease inhibitor cocktail (Sigma-Aldrich). Lysis buffer B, which targets membrane proteins (e.g. HA synthase), was modified from [35] and consists of 50 mM NaH_2_PO_4 _(pH 7), 5 mM EDTA, 4 mg mL^-1 ^lysozyme, 10% (v/v) glycerol and protease inhibitor cocktail (Sigma Aldrich). Cells were then transferred to a disruption tube containing 1.44 g of 100 μm glass beads and lysed using a Mini bead beater (Biospec Products Inc.) In order to prevent samples from overheating, five sets of one minute cycles at 48,000 rpm were performed with samples chilled in ice water for one minute between beatings. Cellular debris was removed by centrifugation at 13,000 × g for 10 min at 4°C and the supernatant stored at -80°C.

### Sample clean-up

Several methods were tested to determine the best precipitation method prior to cell extraction, including a commercial 2-D clean-up (GE-Healthcare), acetone precipitation [45] and TCA/acetone precipitation [46]. After precipitation, proteins were resuspended in lysis buffer A. The protein concentration in the supernatant was determined using the Bradford assay or 2-D Quant kit (GE-Healthcare) according to the manufacturer's instructions.

### Two Dimension electrophoresis

Iso-electric focusing (IEF) was performed using IPG strips (GE-Healthcare). For silver-staining and DIGE, 50 μg or 8 μg of protein were loaded into 24 or 7 cm strips (pH 4–7), respectively. DIGE staining was performed according to manufacturer's instructions using a Typhoon 9410 imager (GE Healthcare) for image analysis. For MS, Coomassie staining was used and 500–800 μg protein was loaded into 24 cm gels by in-gel rehydration. Proteins were separated on an IPGphore I unit (GE, Healthcare) by active rehydration, (30 V) for 12 hrs prior to IEF. IEF was performed according to the following protocol: 1 h, 500 V (Step and hold); 1 h, 1000 V (gradient); 3 hrs, 8000 V (gradient); 12 hrs, 8000 V (Step and hold). After equilibration [47], IPG strips were embedded on top of 12% polyacrylamide gels and electrophoresed in an Ettan Dalt 12 electrophoresis unit (GE Healthcare) at 2 W gel^-1^for 30 min, followed by 18 W gel^-1 ^for 6 hrs. After electrophoresis, gels were fixed in 10% (v/v) acetic acid and 30% ethanol for 1 hr and then stained.

Gel images were analysed using Progenesis SameSpots (Nonlinear dynamics, Newcastle, UK) as described elsewhere [48]. Gels were overlaid by warping and fused to a reference image using the Progenesis algorithm.

### Trypsin digestion, LC-ESI-MS/MS and MALDI-MS-TOF/TOF

Protein spots were excised from the gel and digested with an excess of trypsin (Trypsin Gold, MS grade; Promega) overnight at 37°C as described previously [49]. Peptides were dried using a SpeedVac (SPD111V, Thermo Savant) and redissolved in 80 μL 5% formic acid (aqueous) for MS analysis. An Agilent 1100 Binary HPLC system (Agilent) was used to perform reversed phase separation of the samples prior to MS using a Vydac MS C18 300A, column (150 mm × 2 mm) with a particle size of 5 μm (Vydac). A 30 μL aliquot of sample was injected onto the HPLC column. The mobile phase consisted of solvent A (0.1% aqueous formic acid) and solvent B (90/10 acetonitrile/0.1% aqueous formic acid). Tryptic peptides were eluted using a gradient elution programme of 0–40% B in 80 min, 40–80% B in 10 min and finally a 5 min hold at 80% B, followed by a return to 0% B for a 10-min equilibration. The flow rate was 200 μL min^-1^. Eluate from the RP-HPLC column was directly introduced into the TurboIonSpray source.

Mass spectrometry experiments were performed on a hybrid quadrupole/linear ion trap 4000 QTRAP MS/MS system (Applied Biosystems). The 4000 QTRAP equipped with a TurboIonSpray Source was operated in positive electrospray ionization mode. All analyses were performed using Information Dependent Acquisition and the linear ion trap (LIT) acquisition modes. Analyst 1.4.1 software was used for data analysis. The acquisition protocol used to provide mass spectral data for database searching involved the following procedure: mass profiling of the HPLC eluant using Enhanced Multiple Scan (EMS), ions over the background threshold were subjected to examination using the Enhanced Resolution scan (ER) to confirm charge states of the multiply charged molecular ions. The most and next most abundant ions in each of these scans with a charge state of +2 to +3 or with unknown charge were subjected to CID using rolling collision energy. An enhanced product ion scan was used to collate fragment ions and present the product ion spectrum for subsequent database searches (see details below).

Additionally, some samples were analysed by MALDI-MS using a 4700 Proteomics Analyzer MALDI-TOF/TOF (Applied Biosystems). When necessary, the samples were first desalted using micro C18 ZipTips (Millipore), and peptides eluted directly with 5 mg mL^-1 ^of CHCA in 60% ACN/0.1% aqueous formic acid onto a MALDI target plate. All MS and MS/MS spectra were acquired as reported earlier [50,51] with minor modifications. MS/MS data acquisition was performed in a four-step process. First, MS spectra were recorded from each of the six calibration spots, and the default calibration parameters of the instrument and the plate model for that plate were updated. Secondly, MS spectra were recorded for all sample spots on the plate. Each spectrum was generated by accumulating the data from 1000 laser shots, using the newly updated default calibration settings. Thirdly, the TOF-MS spectra were analyzed using the Peak Picker software supplied with the instrument. The 10 most abundant spectral peaks that met the threshold criteria (>20:1 signal:noise) and were not on the exclusion list were included in the acquisition list for the TOF-TOF, MS/MS portion of the experiment. The threshold criteria were set as follows: mass range: 500 to 4000 Da; minimum cluster area: 500; minimum signal-to-noise (S/N): 20; maximum number of MS/MS spectra per spot: 10. A mass filter excluding matrix cluster ions and trypsin autolysis peaks was applied. An XML file was generated which contained the list of the precursor masses selected for MS/MS.

### Database searching

Database searching of LC-MS/MS, non-interpreted TOF-MS and TOF-TOF MS/MS data was carried out using the ProteinPilot software (version 2.0.1) and Paragon algorithm (Applied Biosystems) in a similar fashion as described elsewhere [52]. The genome of strain ATCC 35246 is not available and we used the published genome for *S. zooepidemicus MGCS10565 *[4] as the main reference. Since *S. zooepidemicus *has been found to be extremely diverse by multilocus sequence typing [53], we obtained the final annotated genome sequence of the H70 strain from the Sanger Institute. A paper describing the H70 genome has been accepted and the genome has been submitted to EMBL (pers. comm., Dr Julian Parkhill). The final sequence without annotation is available from the Sanger Institute .

The MS/MS spectra were extracted and searched against four databases: (i) two protein databases of *S. zooepidemicus *(H70 and MGCS10565), (ii) a modified UniProt database containing the *S. zooepidemicus *genome and (iii) a custom-made database of *S. zooepidemicus *containing randomly shuffled protein entries as a false-positive control. The following search parameters were used: trypsin as enzyme, no Cys modification and the protein confidence threshold cut-off ProtScore 2.0 (unused), with at least two peptides with 99% confidence (see Additional file 2). Finally, PSORT was used to predict protein localization in gram positive bacteria [37].

## List of abbreviations

HA: hyaluronic acid; 2-DE: two-dimensional gel electrophoresis; DIGE: differential gel electrophoresis; IEF: isoelectric focusing; LC: liquid chromatography; MS: mass spectrometry; MALDI: matrix assisted laser desorption/ionization; TOF: time of flight; ESI: electrospray ionisation; DTT: dithiotreitol; IPG: immobilized pH gradient; TCA: trichloroacetic acid; EDTA: ethylenediaminetetraacetic acid; *S.*: *Streptococcus*; UDP: uridine diphosphate; ORF: open reading frames; OD: optical density; Gi: GenInfo Identifier.

## Competing interests

The authors declare that they have no competing interests.

## Authors' contributions

EM did the experimental design, performed 2-DE experiments, optimized 2-DIGE, image analysis, trypsin digestion, contributed to bioinformatics work and participated in the writing of the manuscript. CWG performed protein identification by mass spectrometry and participated in the writing of the manuscript. CA did the genome work including PSORT predictions and participated in the writing of the manuscript. DJC assisted in protein identification and MS and revised the final manuscript. LKN participated in experimental design, data analysis, coordination and preparation of the final version of the manuscript. All authors read and approved the final manuscript.

## Supplementary Material

Additional file 1 **MS/MS spectra were extracted and searched against protein databases based on the MGCS10565 and H70 genomes (see Materials & Methods).** Except for the final column and where indicated by notes, the entries in the table are based on the published, annotated MGCS10565 genome sequence. The final column shows the corresponding locus tag for the H70 genome. Matched peptide sequences for both genomes can be found in "Additional file 2". Click here for file

Additional file 2 **Peptide sequences of identified peptides by ESI LC-MS/MS for strain MGCS10565 (black) and H70 (red).** When the peptide was identified in both genomes it appears in the table in green. Click here for file
