# Supplementary material for: Proteome analysis of the hyaluronic acid-producing bacterium, Streptococcus zooepidemicus
Source: Proteome Sci. 2009 Mar 28;7:13. doi: 10.1186/1477-5956-7-13 (PMC2670282; doi:10.1186/1477-5956-7-13)
Supplement: Additional file 1 — MS/MS spectra were extracted and searched against protein databases based on the MGCS10565 and H70 genomes (see Materials & Methods). Except for the final column and where indicated by notes, the entries in the table are based on the published, annotated MGCS10565 genome sequence. The final column shows the corresponding locus tag for the H70 genome. Matched peptide sequences for both genomes can be found in "Additional file 2". [file 1477-5956-7-13-S1.pdf]

Additional file 1

MS/MS spectra were extracted and searched against protein databases based on the MGCS10565 and H70 genomes (see Materials & Methods). Except for the final column and where indicated by notes, the entries in the table are based on the published, annotated MGCS10565 genome sequence. The final column shows the corresponding locus tag for the H70 genome. Matched peptide sequences for both genomes can be found in additional file 2.

Spot picked	theoretical *pI*	theoretical Mw	protein	Accession number MGCS10565	PSORT prediction	Coding sequence H70
1	4.70	65863.31	chaperone protein DnaK	gi|195977545	cytoplasm	SZO_15780
2	6.49	15828.68	Superoxide dismutase Bos mutus grunniens			
3	5.87	20554.44	ribosome recycling factor Frr	gi|195978553	cytoplasm	SZO_04980
4	5.87	20554.44	3-oxoacyl-[acyl-carrier-protein] reductase FabG	gi|195977553	cytoplasm	SZO_15700
7	5.84	25986.04	ABC transporter ATP-binding protein	gi|195978005	cytoplasm	SZO_10860
9	5.68	23273.92	amino acid transport ATP-binding protein	gi|195978014	cytoplasm	SZO_10770
12	5.64	26293.39	ABC transporter ATP-binding protein	gi|195978242	cytoplasm	SZO_08500
13	5.73	31190.2	exodeoxyribonuclease ExoA	gi|195977651	cytoplasm	SZO_14820
14	5.73	31204.22	glycerol-3-phosphate dehydrogenase (NAD(P)+)	gi|195977364	cytoplasm	SZO_17500
15 [1]	5.68	36617.72	exodeoxyribonuclease ExoA	gi|195978515	cytoplasm	SZO_05480

16	5.34	30295.44	NH(3)-dependent NAD(+) synthetase NadE	gi|195978675	cytoplasm	SZO_03930
17	5.38	35383.39	PTS system mannose-specific EIIAB component ManX	gi|195977564	membrane	SZO_15580
17	5.57	35608.07	enoyl-[acyl-carrier-protein] reductase (FMN)	gi|195977551	membrane	SZO_15720
17	5.22	32357.84	cysteine synthase	gi|195978651	cytoplasm	SZO_04150
18	5.63	48838.48	glutathione reductase	gi|195977995	membrane	SZO_10950
19	5.66	48107.75	seryl-tRNA synthetase SerS	gi|195977559	cytoplasm	SZO_15630
20	5.63	49348.86	bifunctional GcaD protein; bifunctional N-acetylglucosamine-1 -phosphate uridyltransferase / glucosamine-1-phosphate N-acetyltransferase protein GlmU	gi|195978576; gi|195977349	Not Clear	SZO_01730
21	5.55	52925.45	inosine-5'-monophosphate dehydrogenase GuaB	gi|195979041	cytoplasm	SZO_19340
22	5.53	43796.69	3-oxoacyl-[acyl-carrier-protein] synthase II FabF	gi|195977554	cytoplasm	SZO_15690
23	6.67	41256.41	secreted antigen GbpB/SagA/PcsB, putative peptidoglycan hydrolase	gi|195977174	membrane	SZO_00190
24	5.46	45943.1	UDP-N-acetylglucosamine 1-carboxyvinyltransferase MurA	gi|195977930	membrane	SZO_11600
24	5.76	48566.25	UDP-N-acetylglucosamine 1-carboxyvinyltransferase	gi|195977816	cytoplasm	SZO_13180
25	5.55	52925.45	inosine-5'-monophosphate dehydrogenase GuaB	gi|195979041	cytoplasm	SZO_19340
26	5.12	35469.4	PTS system mannose-specific EIIAB component ManX	gi|195977564	cytoplasm	SZO_15580
27	5.38	35383.39	PTS system mannose-specific EIIAB component ManX	gi|195977564	cytoplasm	SZO_15580
28	5.38	35383.39	PTS system mannose-specific EIIAB component ManX	gi|195978008	cytoplasm	SZO_15580
28	5.12	35469.4	6-phosphofructokinase PfkA	gi|195978145	cytoplasm	SZO_10830
30 [2]	5.14	35272.05	L-lactate dehydrogenase	gi|195978145	cytoplasm	SZO_09420

31	5.14	28515.63	30S ribosomal protein S2	gi|195977283	cytoplasm	SZO_01160
31	5.48	34832.65	UTP--glucose-1-phosphate uridylyltransferase HasC.2 ; UTP--glucose-1-phosphate uridylyltransferase 1 HasC.1	gi|195977363; gi|195977348	membrane	SZO_01720
31	5.89	35737.13	UDP-glucose pyrophosphorylase		membrane	
32	5.24	35888.66	NAD-dependent glyceraldehyde-3-phosphate dehydrogenase / plasmin(ogen) receptor	gi|195977417	cytoplasm	SZO_16880
32 [3]	5.09	37139.21	hypothetical protein Sez_1025	gi|195978148	cytoplasm	SZO_09390
33	5.09	37179.23	hypothetical protein Sez_1025	gi|195978148	cytoplasm	SZO_09390
34	4.96	42240.19	phosphoglycerate kinase	gi|195977419	cytoplasm	SZO_16860
34	5.09	44993.05	UDP-glucose 6-dehydrogenase HasB	gi|195977347	membrane	SZO_01710
37	4.76	14222.25	general stress protein	gi|195977498	cytoplasm	SZO_16230
38 4	4.71	8951.21	phosphocarrier protein of PTS system PtsH	gi|195977894	cytoplasm	SZO_11920
39	4.52	26662.11	triosephosphate isomerase TpiA	gi|195978426	membrane	SZO_06470
40 [5,6]	4.83	15756.3	hypothetical protein Sez_1355	gi|195978460	extracellular	SZO_06160
41 [7]	5.07	19593.08	DNA binding protein starved cells-like peroxide resistance protein Dpr	gi|195977723	cytoplasm	SZO_14060
42	5.17	17765.6	single-stranded DNA-binding protein	gi|195977488	cytoplasm	
43	5.30	26140.46	2,3-bisphosphoglycerate-dependent phosphoglycerate mutase GpmA	gi|195977771	cytoplasm	SZO_13630
44 [8]	5.14	20485.22	dTDP-4-Keto-6-Deoxyglucose-3 5-epimerase RmlC	gi|195978346	cytoplasm	SZO_07270
45	5.39	22837.16	peptide deformylase Pdf	gi|195978856	cytoplasm	SZO_02070
46	5.30	26140.46	2,3-bisphosphoglycerate-dependent phosphoglycerate mutase GpmA	gi|195977771	cytoplasm	SZO_13630

46	5.43	25873.56	uridylate kinase	gi|195978554	cytoplasm	SZO_04970
48	4.57	17313.88	ascorbate-specific phosphotransferase enzyme IIA component	gi|195977317	cytoplasm	SZO_01480
49	5.29	19571.33	transcription elongation factor GreA	gi|195978755	cytoplasm	SZO_03180
50	4.82	22477.15	superoxide dismutase [Mn] SodA	gi|195977790	cytoplasm	SZO_13430
50	4.86	23208.74	probable transaldolase	gi|195978702	cytoplasm	SZO_03700
51	4.96	20646.63	hypoxanthine-guanine phosphoribosyltransferase protein Hpt	gi|195977169	cytoplasm	SZO_00140
52	4.98	20128.95	hypothetical protein Sez_0292	gi|195977437	cytoplasm	
54 [9]	4.69	24485.08	ribose-5-phosphate isomerase A RpiA	gi|195978283	cytoplasm	SZO_08070
54	4.63	26791.03	serine/threonine phosphatase PrpC	gi|195978659	cytoplasm	SZO_04080
55	4.60	43876.68	spermidine/putrescine import ATP-binding protein PotA	gi|195978180	cytoplasm	SZO_09070
55	4.68	48332.93	phosphoglucosamine mutase GlmM	gi|195978322; gi|195978068	membrane	SZO_07490
56	4.96	43340.73	S-adenosylmethionine synthetase MetK	gi|195977814	cytoplasm	SZO_13190
56	4.88	49508.87	glucose-6-phosphate isomerase Gpi	gi|195977350	membrane	SZO_01740
57	4.77	47241.27	enolase Eno	gi|195977904	cytoplasm	SZO_11840
57	4.76	47504.96	NADH oxidase H2O-forming Nox	gi|195978146	cytoplasm	SZO_09410
57	4.96	50549.05	aminopeptidase C PepC	gi|195978674	cytoplasm	SZO_03940
57	4.88	49520.93	glucose-6-phosphate isomerase Gpi	gi|195977350	membrane	SZO_01740
58 [10]	4.41	59106.77	dihydroxyacetone kinase family protein	gi|195978812	cytoplasm	SZO_02610
59	4.7	56916.11	heat shock protein 60 family chaperone GroEL	gi|195977293	membrane	SZO_01280

60	4.41	59106.77	dihydroxyacetone kinase family protein	gi|195978812	cytoplasm	SZO_02610
60 [11]	4.7	65863.31	chaperone protein DnaK	gi|195977545	cytoplasm	SZO_15780
61	4.81	32120.68	glucose-1-phosphate thymidylyltransferase RmlA	gi|195978347	cytoplasm	SZO_07260
62	4.84	37933.21	elongation factor Ts	gi|195977282	cytoplasm	SZO_01150
62	4.83	34483.51	DNA-directed RNA polymerase alpha chain	gi|195977234	cytoplasm	SZO_00760
62	4.93	44039.11	aspartate aminotransferase	gi|195977859	membrane	SZO_12250
63	5.57	34792.8	fructose-bisphosphate aldolase Fba	gi|195978815	cytoplasm	SZO_02550
64 [12]	4.86	20608.43	elongation factor P	gi|195977497	cytoplasm	SZO_16240
65	4.95	52307.14	peptidyl-prolyl cis-trans isomerase-like	gi|195978654	cytoplasm	SZO_04130
66	6.55	28229.57	adenylate kinase Adk	gi|195977229	membrane	SZO_00710
67	4.42	16924.29	biotin carboxyl carrier protein of acetyl-CoA carboxylase AccB	gi|195977555	cytoplasm	SZO_15680
68	4.67	13902.01	hypothetical protein Sez_0439	gi|195977580	cytoplasm	SZO_15420
69	4.52	20511.3	transcription antitermination protein NusG	gi|195977307	cytoplasm	SZO_01410
70	4.56	28845.89	cell division initiation protein DivIVA	gi|195977737	cytoplasm	SZO_13910
71	5.14	28013.99	30S ribosomal protein S2	gi|195977283	cytoplasm	SZO_01160
72	5.14	34909.56	extracellular protein	gi|195978139	extracellular	SZO_09490
72	4.7	32069.35	transcription regulator	gi|195978835	cytoplasm	SZO_02390
74	4.83	22478.18	superoxide dismutase [Mn] SodA	gi|195977790	cytoplasm	SZO_13430
76 [13]	4.68	24515.10	rpiA ribose 5-phosphate somerise A		cytoplasm	SZO_08070

77 [14]	4.57	46776.18	ftsZ cell division protein FtsZ		cytoplasm	SZO_13960
78	4.88	43868.59	elongation factor Tu TuF	gi195978427	cytoplasm	SZO_06460
80	5.09	44975.01	UDP-glucose 6-dehydrogenase HasB	gi195977347	membrane	SZO_01710
81	5.48	34832.65	putataive 3-oxoacyl-[acyl-carrier-protein] synthase III	gi195977549	membrane	SZO_15740
82	4.45	47172.79	cell division trigger factor Tig	gi:195978819	membrane	SZO_02510

Additional file subscripts

gi: GenInfo Identifier

[1] The same protein is called ccpA catabolite control protein in H70

[2] rpsB 30S ribosomal protein S2 was found in H70 for this spot.

[3] rmlB putative dTDP-glucose-4,6-dehydratase H70 for this spot.

[4] rplN 50S ribosomal protein L14 found in H70 for this spot.

[5] Annotated as a putative exported protein in H70 for this spot.

[6] A conserved hypothetical protein, SZO_16661 was also found for this spot in H70.

[7] rplJ 50S ribosomal protein L10 was found in H70 for this spot.

[8] rpsD 30S ribosomal protein S4 was also found in H70 for this spot.

[9] rplC 50S ribosomal protein L3 was also found in H70 for this spot.

[10] Annotated as dihydroxyacetone kinase family protein in MGCS10565 and putative phosphatase in H70. A blast-p of the sequences picks dihydroxyacetone kinase, which is most likely to be the correct annotation.

[11] Annotated as chaperone protein DnaK and putative phosphatase in H70. A blast-p of the sequences picks Dak-2 superfamily, which is most likely to be the correct annotation.

[12] 30S ribosomal protein S4 was also found in H70 for this spot.

[13] Protein found only in the H70 genome.

[14] Protein found only in the H70 genome.
